# Acetylcholinesterase-Inhibiting Activity of Salicylanilide *N*-Alkylcarbamates and Their Molecular Docking

**DOI:** 10.3390/molecules170910142

**Published:** 2012-08-24

**Authors:** Ales Imramovsky, Sarka Stepankova, Jan Vanco, Karel Pauk, Juana Monreal-Ferriz, Jarmila Vinsova, Josef Jampilek

**Affiliations:** 1 Institute of Organic Chemistry and Technology, Faculty of Chemical Technology, University of Pardubice, Studentska 573, 532 10 Pardubice, Czech Republic; 2 Department of Biological and Biochemical Sciences, Faculty of Chemical Technology, University of Pardubice, Studentska 573, 532 10 Pardubice, Czech Republic; 3 Department of Chemical Drugs, Faculty of Pharmacy, University of Veterinary and Pharmaceutical Sciences Brno, Palackeho 1/3, 612 42 Brno, Czech Republic; 4 Department of Inorganic and Organic Chemistry, Faculty of Pharmacy in Hradec Kralove, Charles University in Prague, Heyrovskeho 1203, 500 05 Hradec Kralove, Czech Republic

**Keywords:** 4-chloro-2-(chlorophenylcarbamoyl)phenyl alkylcarbamates, *in vitro* acetyl-cholinesterase inhibition, lipophilicity, molecular docking

## Abstract

A series of twenty-five novel salicylanilide *N*-alkylcarbamates were investigated as potential acetylcholinesterase inhibitors. The compounds were tested for their ability to inhibit acetylcholinesterase (AChE) from electric eel (*Electrophorus electricus* L.). Experimental lipophilicity was determined, and the structure-activity relationships are discussed. The mode of binding in the active site of AChE was investigated by molecular docking. All the discussed compounds expressed significantly higher AChE inhibitory activity than rivastigmine and slightly lower than galanthamine. Disubstitution by chlorine in C'_(3,4)_ of the aniline ring and the optimal length of hexyl-undecyl alkyl chains in the carbamate moiety provided the most active AChE inhibitors. Monochlorination in C'_(4)_ exhibited slightly more effective AChE inhibitors than in C'_(3)_. Generally it can be stated that compounds with higher lipophilicity showed higher inhibition, and the activity of the compounds is strongly dependent on the length of the *N*-alkyl chain.

## 1. Introduction

It is well-known that salicyl-like compounds show ability to inhibit cyclooxygenase (COX) [1]. Various carbamates are also known as reversible cholinesterase inhibitors [1]. Salicylanilides (2-hydroxy-*N*-phenylbenzamides) and/or their *O*-substituted derivatives show diverse pharmacological activities [2,3,4,5,6,7,8]. The presence of the amide group (-NHCO-) together with the carbamate group (-NHCOO-) in the structure of phenylcarbamoylphenyl *N*-alkylcarbamates can cause interaction with various enzymes or enzymatic systems, e.g. both amide and carbamate moieties are characteristic for a number of herbicides acting as photosynthetic electron inhibitors [7,8,9], and the carbamate moiety or its bioisosteres are typical for a number of potential acetylcholinesterase inhibitors [10,11,12,13,14,15,16,17,18,19].

Acetylcholinesterase (AChE, EC 3.1.1.7) plays a vital role in the central and peripheral nervous systems, where it catalyzes the hydrolysis of the neurotransmitter acetylcholine (ACh) in cholinergic synapses, and subsequently it can affect a number of pathogenic processes [16]. AChE inhibitors are used in treatment of various neuromuscular disorders and have provided the first generation of drugs for treatment of Alzheimer’s disease (AD) [20], which is a progressive physical disorder that causes increasingly severe impairment in the cognitive and functional ability of individuals suffering from the disease [21]. The progression of this neurodegenerative disease leads to dementia [22]. The cause for most Alzheimer’s cases is still essentially unknown. Several competing hypotheses exist trying to explain the cause of the AD [23]. The oldest, on which most currently available drug therapies are based, is the cholinergic hypothesis that proposes that AD is caused by reduced synthesis of the neurotransmitter ACh [24]. This cholinergic hypothesis has not maintained widespread support, nevertheless it can be stated that the cholinergic deficit is responsible for the symptomatology of AD [23]. The inhibition of AChE causes an increase in the concentration of ACh in cholinergic synapses, which results in alleviation of the disease. For this reason, new and potent AChE inhibitors may be helpful in the treatment of this disease.

*N*-Substituted carbamates are characterized as pseudosubstrate inhibitors or active site-directed irreversible inhibitors of AChE that, in the presence of substrate, compete for the AChE active site. A proposed simplified mechanism for AChE inhibition by carbamates consists in rapid formation of the covalent enzyme-inhibitor tetrahedral intermediates via a nucleophilic attack of the hydroxyl moiety in Ser200 of the enzyme to the carbamate carbonyl and following slow generation of a carbamylated-enzyme intermediate from the tetrahedral intermediate. The hydroxyl-moiety of the carbamate ester can be consequently recovered at a very slow rate or blocked irreversibly depending on the character of the used *N*-substituted carbamates [1,14,15,16,17,18,19].

Reaching the inhibitory site of action and binding to the active site gorge would be influenced by hydro/lipophilic properties of the compounds; therefore the lipophilicity of the target compounds was experimentally determined using RP-HPLC. Structure-activity relationships between the chemical structure, physical properties and biological activities of the evaluated compounds are discussed. The specific orientation of the inhibitors in the AChE binding site was suggested using molecular docking.

## 2. Result and Discussion

### 2.1. Chemistry

**Scheme 1:**
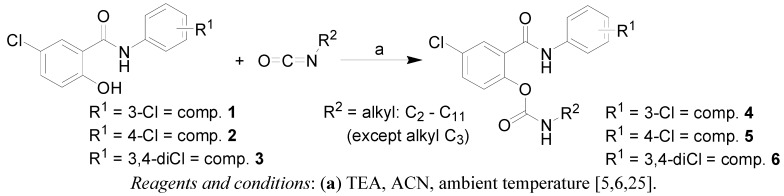
Synthesis of target 4-chloro-2-(chlorophenylcarbamoyl)phenyl alkylcarbamates **4**, **5** and **6**.

The synthesis of the discussed carbamates is shown in Scheme 1. The synthetic pathway was discussed recently [6,25]. The starting salicylanilides **1**–**3** were routinely prepared by the reaction of 5-chlorosalicylic acid with the appropriate aniline and PCl_3_ in chlorobenzene using a microwave reactor [3]. For the synthesis of the corresponding *N*-alkylcarbamates **4a**–**6i**, a suspension of salicylanilides **1**–**3** in acetonitrile (ACN) was treated with equivalent of triethylamine (TEA), followed by adding of the appropriate isocyanate. This reaction was performed at ambient temperature due to thermal instability of the products. The prepared *N*-alkylcarbamates **4a**–**6i** belong to three groups: those having chlorine at positions C'_(3)_, C'_(4)_ and C'_(3,4)_ of the anilide ring; the prepared compounds are summarized in Table 1. The yields of the synthesized compounds varied in the interval of 35–80%. A small library of discussed compounds **4a**–**6i** was characterised by means of IR, NMR spectroscopy, CHN analysis and published recently [6,8]. The chemical stability (carbamate bond hydrolysis and the release of the parent salicylanilide) of the discussed compounds were studied in aqueous buffer solutions at pH 3–8 at 37 °C by HPLC. The compounds were stable at acidic pH values, but they were decomposed in alkaline environment, approx. T_1/2_ 64 h at pH 7.0 and approx. T_1/2_ 43 h at pH 7.4 were shown [6].

### 2.2. Lipophilicity

Lipophilicity is a property that has a major effect on absorption, distribution, metabolism, excretion and toxicity properties as well as pharmacological activity, because drugs cross biological membranes through the passive transport, which strongly depends on their lipophilicity. Lipophilicity has been studied and applied as an important drug property for decades. Different lipophilicity descriptors such as log *k_w_*, log *P*, log *D*, *etc*. are used for structure-activity relations description and prediction. However, the algorithms used in their calculation do not take into account configuration specificity and sometimes even intramolecular interactions of these *N*-substituted carbamoyl derivatives.

**Table 1:** IC_50_ Values of AChE inhibition in comparison with standards rivastigmine (RIV) and galanthamine (GLT) and lipophilicity values (log *k*, log *P*). AChE inhibition is expressed as mean ± SD (*n* = 3 experiments), (N.D. = not determined; * calculated by ACD/LogP (ver. 1.0), Advanced Chemistry Development Inc., Toronto, Canada).

Comp.		R ^1^	R ^2^	log *k*	log *P* *	AChE IC_50_ (μmol/L)
**GROUP 1**	**4a**	3-Cl	C_2_H_5_	0.7523	4.02 ± 0.40	N.D.
	**4b**	3-Cl	C_4_H_9_	0.7611	5.08 ± 0.40	N.D.
	**4c**	3-Cl	C_5_H_11_	0.7732	5.61 ± 0.40	50.4 ± 0.48
	**4d**	3-Cl	C_6_H_13_	0.7758	6.14 ± 0.40	35.1 ± 0.31
	**4e**	3-Cl	C_7_H_15_	0.7772	6.67 ± 0.40	61.7 ± 0.50
	**4f**	3-Cl	C_8_H_17_	0.7831	7.20 ± 0.40	69.9 ± 0.49
	**4g**	3-Cl	C_9_H_19_	0.7837	7.74 ± 0.40	34.2 ± 0.26
	**4h**	3-Cl	C_10_H_21_	0.7873	8.27 ± 0.40	52.7 ± 0.45
	**4i**	3-Cl	C_11_H_23_	0.7902	8.80 ± 0.40	56.9 ± 0.48
**GROUP 2**	**5a**	4-Cl	C_2_H_5_	0.7695	3.98 ± 0.39	59.1 ± 0.33
	**5b**	4-Cl	C_4_H_9_	0.7759	5.04 ± 0.39	48.1 ± 0.31
	**5c**	4-Cl	C_5_H_11_	0.7838	5.57 ± 0.39	41.7 ± 0.29
	**5d**	4-Cl	C_6_H_13_	0.7859	6.10 ± 0.39	31.0 ± 0.29
	**5e**	4-Cl	C_7_H_15_	0.7872	6.63 ± 0.39	49.4 ± 0.31
	**5f**	4-Cl	C_8_H_17_	0.7899	7.16 ± 0.39	62.5 ± 0.41
	**5g**	4-Cl	C_9_H_19_	0.7919	7.70 ± 0.39	52.2 ± 0.39
	**5h**	4-Cl	C_10_H_21_	0.7942	8.23 ± 0.39	64.6 ± 0.41
	**5i**	4-Cl	C_11_H_23_	0.7947	8.76 ± 0.39	44.3 ± 0.33
**GROUP 3**	**6a**	3,4-Cl	C_2_H_5_	1.0234	4.88 ± 0.41	67.3 ± 0.52
	**6b**	3,4-Cl	C_4_H_9_	1.0457	5.94 ± 0.41	64.7 ± 0.79
	**6c**	3,4-Cl	C_5_H_11_	1.0601	6.47 ± 0.41	56.0 ± 0.42
	**6d**	3,4-Cl	C_6_H_13_	1.0611	7.01 ± 0.41	41.2 ± 0.36
	**6e**	3,4-Cl	C_7_H_15_	1.0667	7.54 ± 0.41	36.7 ± 0.28
	**6f**	3,4-Cl	C_8_H_17_	1.0845	8.07 ± 0.41	32.1 ± 0.29
	**6g**	3,4-Cl	C_9_H_19_	1.0880	8.60 ± 0.41	43.9 ± 0.32
	**6h**	3,4-Cl	C_10_H_21_	1.0894	9.13 ± 0.41	21.8 ± 0.20
	**6i**	3,4-Cl	C_11_H_23_	1.0903	9.66 ± 0.41	29.9 ± 0.22
**RIV**		–	–	–	2.14 ± 0.27	501 ± 3.08
**GLT**		–	–	–	1.59 ± 0.45	4.0 ± 0.13

Calculation of log *P*/Clog *P* by CS ChemOffice Ultra ver. 10.0 is based on references [26,27,28,29,30,31], not in order while calculation of log *P* by ACD/LogP ver. 1.0 is based on Hansch and Leo [32]. The program ChemOffice did not resolve various lipophilicity values of individual positional isomers, in particular, the same log *P*/Clog *P* data were calculated for series 3-Cl (compounds **4a**–**i**) and series 4-Cl (compounds **5a**–**i**), therefore these data are not included in Table 1. Log *k* data specify lipophilicity within the series of the discussed compounds, and all the chiral compounds were measured several times with the same results. The determined differences in log *k* parameters for individual *R*/*S*-enantiomers cannot be explained on the basis of the results presented here.

### 2.3. *In Vitro* Evaluation of AChE-Inhibiting Activity

The discussed compounds were tested for their ability to inhibit AChE from electric eel (*Electrophorus electricus* L.) using Ellman’s method [12,13], and they expressed good activity compared with the drugs in clinical practice rivastigmine (Exelon^®^) and galanthamine (Reminyl^®^). All the discussed carbamate-like compounds expressed significantly higher AChE inhibitory activity than the standard rivastigmine and slightly lower inhibitory activity than the standard galanthamine. The IC_50_ values are summarized in Table 1.

The compounds under investigation could be divided into three groups based on their substitution: *Group 1* includes 3-Cl substituted carbamates **4a**–**i**; *Group 2* contains 4-Cl derivatives **5a**–**i**; and *Group 3* is composed of 3,4-Cl disubstituted compounds **6a**–**i**, see Table 1. The most potent AChE inhibition of the isolated enzyme was observed for *Group 3* (C'_(3,4)_ series) that provided the most active inhibitors: compounds **6h** (R^2^ = decyl, IC_50_ = 21.8 µmol/L, 23-fold higher than RIV) and **6i** (R^2^ = undecyl, IC_50_ = 29.9 µmol/L, 16-fold higher than RIV). Monochlorination of C'_(3)_ provided less effective inhibitors than C'_(4)_ monosubstitution: compound **5d** (R^2^ = hexyl, IC_50_ = 31.0 µmol/L). These results demonstrated that 4-chloro-2-(3,4-chlorophenylcarbamoyl)phenyl long-chain-*N*-alkylcarbamates can interact with all the binding AChE sites more tightly than the monochlorinated compounds and block the AChE gorge.

**Figure 1:**
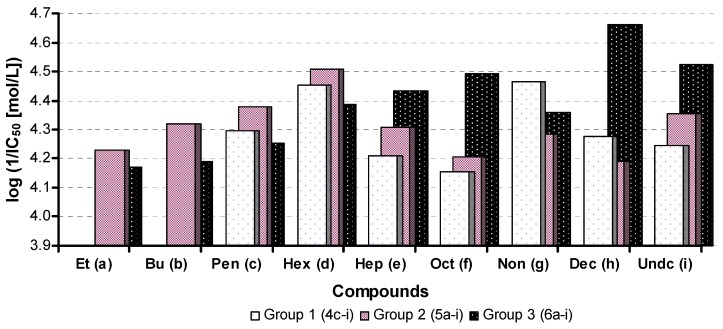
Dependence of AChE inhibition (log 1/IC_50_ [mol/L]) on long alkyl chain of R^2^ substitution within individual *Groups 1*–*3*. (Et = ethyl, Bu = butyl, Pen = pentyl, Hex = hexyl, Hep = heptyl, Oct = octyl, Non = nonyl, Dec = decyl, Undc = undecyl).

**Figure 2:**
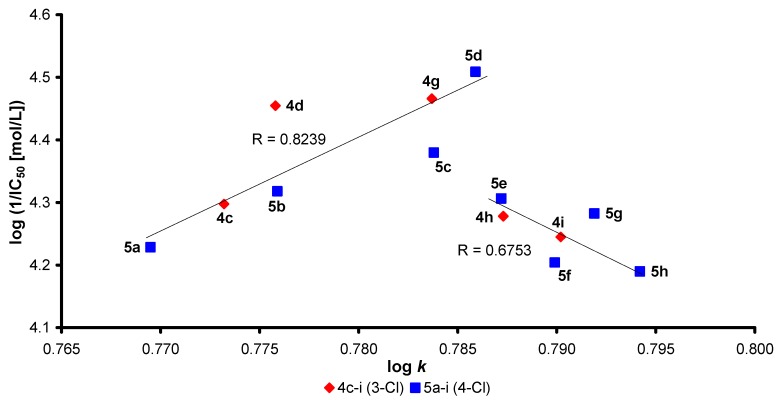
General dependences of AChE inhibition (log 1/IC_50_ [mol/L]) on lipophilicity of compounds **4** and **5** expressed as log *k*. Compounds **4e**, **4f** and **5i** were excluded/are not plotted.

**Figure 3:**
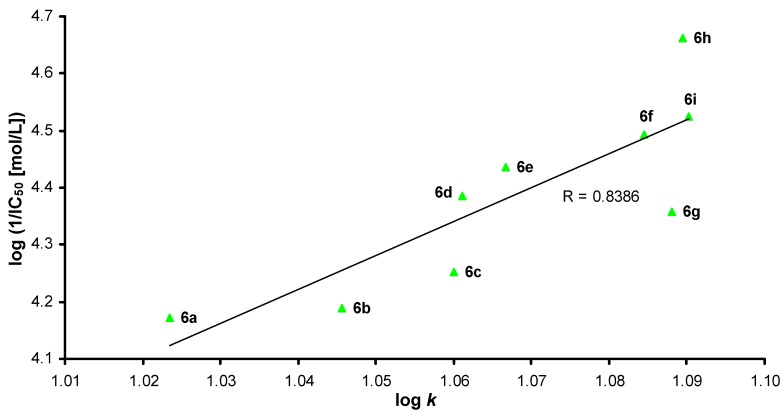
Dependence of AChE inhibition (log 1/IC_50_ [mol/L]) on lipophilicity of compounds **6** expressed as log *k*.

The dependences of AChE inhibition (1/IC_50_) on R^2^ substitution (the length of the alkyl chain) of the discussed compounds are illustrated in Figure 1. A similar trend as in Figure 1 can be observed for dependences of AChE inhibition on compound lipophilicity expressed as log *k*, see Figure 2 and Figure 3. Generally it can be stated that compounds with higher lipophilicity, that is caused by 3,4-Cl disubstitution, are more effective inhibitors, *Group 3* (**6a**–**i**) >>> *Group 2* (**5a**–**i**) > *Group 1* (**4c**–**i**). Within monochlorinated compounds different dependences compared with dichlorinated carbamates can be found, see Figure 2 and Figure 3. Thus it is evident that activity is dependent both on the length of the alkyl chain (R^2^ substituent) and on lipophilicity.

The shapes of biological response curves shown in Figure 1 are typical dependences of *n*-alkyl homologous series, when activity can alternate with the number of carbon atoms (with a change of a long side/straight chain) resulting in a “zigzag” pattern. Inhibition ability of carbamates (binding to the active site of AChE) seems to be influenced by the length of alkyl substituents due to the difficulty of larger inhibitors to enter the narrow enzyme active site gorge as well as strictly balanced hydro/lipophilic properties of the inhibitors, taking into account the fact that hydrophobic substituents accelerate the inhibitors entering the active site gorge of the enzyme [15]. Further, Davis *et al*. also described that long-chain *N*-alkylcarbamate AChE inhibitors are much more stable than their short-chain homologues [33].

A number of papers refer to the fact that an increase of *N*-alkyl chain length resulted in AChE inhibition, especially to *N*-hexyl. A further increase of the chain length resulted in a great variation in carbamylation rates. This fact is in compliance with observation that the introduction of bulky hydrophobic groups at the carbamoyl nitrogen led to compounds with better AChE inhibition [15,17,19,34]. It was also observed that within the series of phenyl *N*-methyl carbamates activity increases with increasing electron-withdrawing effect [10]. From these facts it can be concluded that AChE inhibition can be correlated with hydrophobicity, electronic, inductive or polar properties and steric effects [15].

Monochlorinated carbamates C'_(3)_ (*Group 1*, **4**) or C'_(4)_ (*Group 2*, **5**) expressed different dependences in comparison with C'_(3,4)_ dichlorinated carbamates (*Group 3*, **6**). Series **4** and **5** possess similar log *k* values (differ from each other by about 1%) compared with C'_(3,4)_ series **6**. *Group 1* differs from *Group 2* only by chlorine position in the anilide part; therefore some comparable trends/shapes can be observed. In both cases AChE inhibition grows exponentially to derivatives **4d**, **5d** (R^2^ = hexyl, IC_50_ = 35.1, 31.0 µmol/L), then decreases to compounds **4f**, **5f** (R^2^ = octyl, IC_50_ = 69.9, 62.5 µmol/L), after that increases to carbamates **4g**, **5g** (R^2^ = nonyl, IC_50_ = 34.2 or 52.2 µmol/L) and subsequently decreases again. Only in *Group 2* (C'_(4)_) activity increases insignificantly to compound **5i** (R^2^ = undecyl, IC_50_ = 44.3 µmol/L). This fact can be connected with probably different fitting of C'_(4)_ chloro substitution in comparison with carbamates group C'_(3)_ substituted with chlorine, but in general it can be stated that both series expressed similar AChE inhibition. Two compounds **4d** and **4g** showed similar activities, their lipophilicity (log *k*) is approaching to the log *k* value of compound **5d**, and further lipophilicity increase (alkyl chain prolongation) did not have any influence on AChE inhibition increase. Therefore lipophilicity seems to be an important parameter. These results can be explained by the fact that the optimal lipo/hydrophilic ratio for monosubstituted *N*-alkylcarbamates is about 0.786, and hexyl or nonyl chains are flexible and both can sterically fit the active site of the AChE gorge.

When from the set of 16 tested compounds of *Group 1* and *Group 2* the least active carbamates/outlier, *i.e.*, **4f**, **4e** and **5i**, were eliminated, the dependences of AChE inhibition (log 1/IC_50_, [mol/L]) on log *k* can be presented in Figure 2. In the first case (to log *k* ca 0.786, C_6_ for **5d** or C_9_ for **4g**) the dependence of activity on lipophilicity is linear with correlation coefficient R = 0.8239. As discussed above, the activity showed a “zigzag” pattern, and the range of individual inhibition values is narrow, therefore in the second case the dependence of AChE inhibition of significantly less effective carbamates **4h**, **4i**, **5e**, **5f**, **5g** and **5h** on lipophilicity (log *k* from ca 0.787) does not seem to be so clear, but generally, for *Group 1* and *Group 2*, it can be stated that AChE inhibition dramatically decreases with lipophilicity increase. This observation supported the above mentioned fact that activity is dependent both on the length of the alkyl chain and on lipophilicity (substitution of anilide ring), then on the balanced lipo/hydrophilic properties (lipophilicity/polarity) of the individual discussed carbamates due to their optimal interaction with the active site of the AChE gorge.

Different AChE inhibition results can be observed for *Group 3*. In general, the whole series **6** showed higher activity than both monochlorinated series **4** and **5**. The lipophilicity of the compounds within *Group 3* is much higher than within *Group 1* and *Group 2*, as mentioned above, and the optimal lipo/hydrophilic ratio for disubstituted *N*-alkylcarbamates is about 1.089. Inhibition capability grows exponentially to the local maximum, compound **6f** (R^2^ = octyl, IC_50_ = 32.1 µmol/L), which is similar to the above mentioned most effective chlorine monosubstituted compounds **4d**, **4g** and **5d**. After that it decreases to derivative** 6g** (R^2^ = nonyl, IC_50_ = 43.9 µmol/L), then significantly grows to the most active compound **6h** (R^2^ = decyl, IC_50_ = 21.8 µmol/L) and slightly decreases to carbamate **6i** (R^2^ = undecyl, IC_50_ = 29.9 µmol/L), which is the second effective compound among all the discussed *N*-alkylcarbamates. The dependence of AChE inhibition (log 1/IC_50_, [mol/L]) on log *k* of compounds from *Group 3* is illustrated in Figure 3. The approximately linear dependence with correlation coefficient R = 0.8386 of AChE inhibition on lipophilicity of compounds **6** can be observed.

This different AChE-inhibiting behaviour of C'_(3,4)_ dichlorinated series **6** can be probably explained based on the easier entrance to the gorge and slightly different fitting of these carbamates to the active site of AChE (different docking to the active site due to both chlorine atoms in the anilide phenyl). Higher inhibition ability of C'_(3,4)_ dichlorinated derivatives may be also partly caused by the increase of electron-withdrawing effect (Hammett’s σ parameters) of individual substituents in the anilide part of the molecule [35]. As discussed above, inhibition potential of the carbamates is also affected by the bulk parameter. The variations in the chain length exhibited an obvious effect on AChE inhibition. Generally, within monosubstituted *Groups 1* and *2* C_6_ and/or C_9_ expressed the highest AChE inhibitory activity, C_8_ derivative showed the lowest inhibition, and the activities of *N*-alkylated C_5_, C_7_, C_10_ and C_11_ carbamates were almost equal but lower in comparison with disubstituted *Group 3*, where C_10_, C_11_ and C_8_ expressed the highest AChE inhibitory activity, and inhibition of *N*-alkylated C_6_, C_7_ and C_9_ carbamates was almost the same but lower.

### 2.4. Molecular Docking

Structural analysis of AChE revealed that the active site is placed near the bottom of a narrow gorge imbedded halfway into the protein and 14 aromatic residues lining a substantial portion of the surface of the gorge. This cavity was named the ‘active site gorge’ and, further at the gorge mouth a peripheral anionic binding site (PAS) was found. The active site of AChE contains: (1) an esteratic site (ES) comprising the catalytic triad Ser200-His440-Glu327, which is located at the bottom of the gorge; (2) an oxyanion hole (OAH) that stabilizes the tetrahedral intermediate binding of the carbamate carbonyl group; (3) an acyl binding site (ABS) that binds the acetyl group of ACh or the alkyl moiety of carbamate inhibitors; (4) an anionic substrate binding site (AS) that contains a small number of negative charges but many aromatic residues, where the quaternary ammonium pole of ACh and of various active site ligands binds through a preferential interaction of quarternary nitrogens or a partial positive charge generated by electron-withdrawing moieties.

Structural analysis of the *Torpedo californica* AChE demonstrated an active site near the bottom of a 20 Å deep and narrow gorge imbedded halfway into the protein and 14 aromatic residues lining a substantial portion of the surface of the gorge. This cavity was named the ‘active site gorge’, and at the gorge mouth a peripheral anionic binding site (PAS) was found. The active site of AChE contains: (1) an esteratic site (ES) comprising the catalytic triad Ser-His-Glu, which is located at the bottom of the gorge; (2) an oxyanion hole (OAH) that stabilizes the tetrahedral intermediate binding of the carbamate carbonyl group; (3) an acyl binding site (ABS) that binds the acetyl group of ACh or the alkyl moiety of carbamate inhibitors [36]; (4) an anionic substrate binding site (AS) that contains a small number of negative charges but many aromatic residues, where the quaternary ammonium pole of ACh and of various active site ligands binds through a preferential interaction of quarternary nitrogens or a partial positive charge generated by electron-withdrawing moieties. The distance between the ES and the AS is 5 Å [11,15,37].

Due to these facts, carbamate inhibitors would be the most efficient if the distance between the ES and the AS is also 5 Å, therefore *meta*-carbamates are more advantageous. Nevertheless Sundberg *et al.* stated that compounds with a non-rigid skeleton (cycles linked with flexible chains) could adapt to the binding site and are not necessarily constrained to the extended structure present in the crystal, e.g., various *ortho*-carbamate inhibitors were described [11,14,38,39], or the differently substituted *N*-alkylcarbamylphloroglucinols with high rotational freedom of long-chain substituents were described in the recent paper of Lin *et al.* [36]. In addition to the majority of carbamate inhibitors binding to the active site of AChE and blocking the hydroxyl moiety in Ser in the ES, dual-site binding inhibitors of AChE were described recently, e.g. donepezil. These dual-site binding inhibitors bind to both the active and the peripheral sites, especially Trp in the PAS [40,41,42,43]. Further different dual or multiple-site inhibitors of AChE were prepared and showed stacking interaction with Trp or Tyr in the PAS or with Tyr and Phe that existed in the middle of the gorge [11,44,45,46].

**Figure 4:**
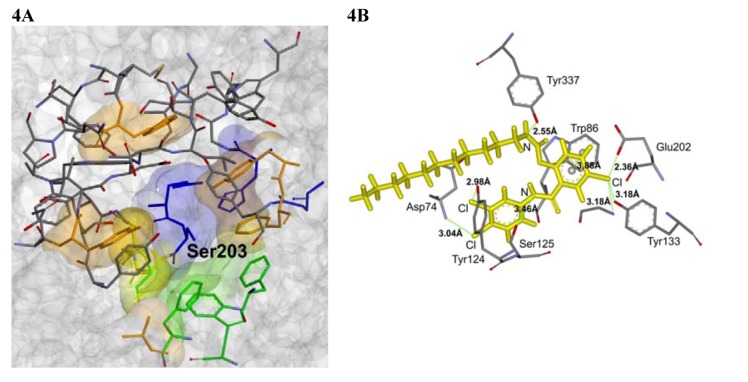
(**A**) Representation of three-dimensional structure of acetylcholinesterase (shown as solvent accessible surface), showing gorge and specific parts of active site (catalytic triad: blue, OAH: yellow, PAS: orange, ABS: green). (**B**) Example of interactions between docked molecule of **6i** and amino acid residues within active site gorge. The interacting atoms and amino acid residues are depicted; other parts of the active site gorge are omitted for clarity.

**Figure 5:**
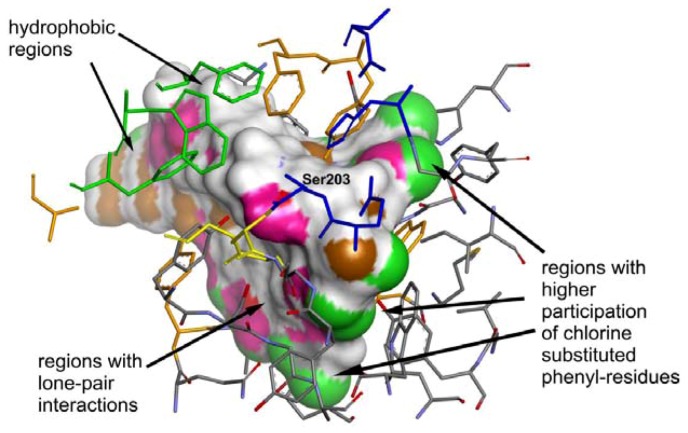
Three-dimensional view from within active site gorge (using same colour scheme for active site as in Figure 4A), containing the overlaid highest-ranked conformations of all docked ligands, represented by solvent accessible surface coloured by element appearance (chlorine-green, carbon and hydrogen-brown or white) or lone pair presence (purple colour), showing regions with preferred orientation of different residues (especially hydrophobic region near ABS site, regions containing high occurrence of lone-pair interactions near OAH and PAS, and regions with higher occurrence of chlorine-substituted phenyl residues near catalytic triad).

Molecular docking within the study was carried out using Gold 5.0 (CCDC, Cambridge, UK) to produce the optimal conformations for the docked ligands. The set of best ranked conformations of the discussed compounds can be characterized, similarly as by Paz *et al*. [46], by low direct interaction with the catalytic triad. On the other hand, they bind to the peripheral part of the gorge by means of the hydrogen bonds between the amide nitrogen and the Ser125 side chain (O-N = 3.226–3.465 Å) and between the amide oxygen and the Ser125 side chain (O-O = 3.969–4.222 Å) or Gly126 nitrogen (N-O = 3.897 Å). Interactions of the carbamate nitrogen with the Tyr337 OH moiety (N-O = 2.553–2.711 Å) and π-π interaction between the trisubstituted phenyl ring and the Trp86 side chain (centroid-centroid = 3.875–3.957 Å) were approved as real. The interactions Cl··H-N/O that were identified in the molecular docking experiments could also play an important role in positioning of the ligands inside the active site gorge. Interestingly, in accordance with the recent paper from Lin *et al.* [36], the conformation of carbamate residue observed for the most active inhibitors was found to be in *cis*-arrangement which allowed the molecules to form the “hairpin”-like structure. On the other hand, in comparison with Lin *et al.* [36], the carbamate residue in the tested compounds is not involved in the interactions with the catalytic triad of the active site gorge. This could be due to the differences in constitution of the derivatives described by Lin *et al.* [36] and the compounds presented in our manuscript, while the first ones contain only one part of higher rigidity (*i.e.*, the aromatic system of differently substituted benzene ring) and all other parts have high freedom of rotation. The compounds described in our manuscript, on the other hand, contain semi-rigid residue of two substituted benzene ring connected by amide group and only one freely rotable alkyl-carbamate chain. The representative example of interaction of the active AChE-inhibitor **6i** and the amino acid residues within the active site gorge is depicted at Figure 4. The low-energy conformations of the investigated compounds (especially the most active derivatives involving the long alkyl-chain substitution) confirmed that the molecule polar parts are oriented to the catalytic gorge and non-polar parts, to the PAS. The graphical model of the AChE molecule involving three most active AChE inhibitors (**6h**, **6i** and **5d**) and demonstrating the conformations of docked ligands is shown in Figure 5. This inhibitor orientation can cause a competitive reaction with native ionic ligand, considerable ACh repulsion and resulting effective AChE blocade.

In case of the studied protein, the gorge of AChE contains the catalytic triad (Ser203, His447, Glu334), marked blue in Figure 4A and other parts that are used to correct the orientation of the normal substrate (ionized acetylcholine) within the gorge like “oxanion hole” (OAH) formed by Ala204, Gly121 and Gly122 (indicated by yellow colour in Figure 4A), which is used to correct the orientation of the ester group of acetylcholine. Another specific site is called “peripheral anionic binding site” (PAS), serves to orient the cationic part of acetylcholine, is located in the peripheral part of the gorge and is composed of Trp86, Tyr337, Trp286 and Tyr72 (marked by orange colour in Figure 4A). The space for acetyl function is provided by “acyl binding site” (ABS), which is located near the OAH and is formed by Trp236, Phe338, Phe295, Phe297 and Gly122 (marked by green colour in Figure 4A).

**Figure 6:**
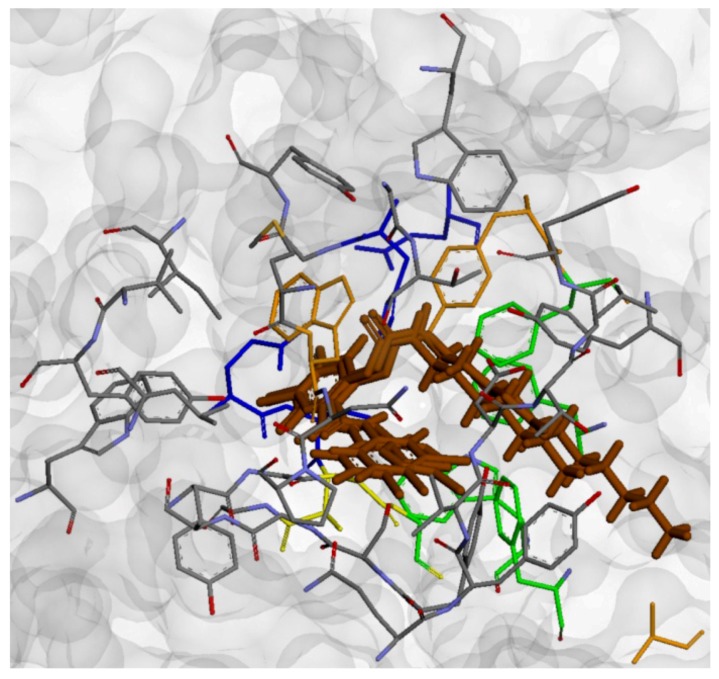
Graphical representation of AChE structure (solvent accessible surface, PDB ref. 2WHQ) with highlighted active site and three best ranked conformations of AChE inhibitors (**6h**, **6i** and **5d**, marked by brown color).

The molecular docking yielded a series of conformations of ligands, see Figure 5, that represented quite different modes of interactions, dependent on their structure. The derivatives involving short lipophilic substitution usually folded deep within the gorge, near the catalytic triad and OAH. On the other hand, the highly active derivatives involving longer lipophilic substitution (6 to 11 carbon long alkyl chain) interacted mainly near the OAH and folded throughout the gorge, exiting it with the linear alkyl chain near the most lipophilic part of the gorge (near the ABS), see Figure 6. On the basis of such orientation and the set of interactions, which were identified between the ligands and the protein residues within the gorge, it can be hypothesized that the studied compounds could act as “bulky”-blockers of the normal ionic substrate (ACh) entrance into the active site gorge.

## 3. Experimental

### 3.1. Chemistry

Synthesis and characterization of the discussed 4-chloro-2-{[(3-chlorophenyl)amino]carbonyl} phenyl alkyl-carbamates (**4a**–**i**), 4-chloro-2-{[(4-dichlorophenyl)amino]carbonyl}phenyl alkyl carbamates (**5a**–**i**) and 4-chloro-2-{[(3,4-dichlorophenyl)amino]carbonyl}phenyl alkylcarbamates (**6a**–**i**) were described by Ferriz *et al.* [6] and Imramovsky *et al.* [8,25] and characterization of all the prepared compounds was recently reported [6].

### 3.2. Lipophilicity Determination by HPLC (Capacity Factor k/Calculated log k)

A Waters Alliance 2695 XE HPLC separation module and a Waters Photodiode Array Detector 2996 (Waters Corp., Milford, MA, USA) were used. A Symmetry^®^ C_18_ 5 μm, 4.6 × 250 mm chromatographic column, Part No. WAT054275 (Waters Corp., Milford, MA, USA) was used. The HPLC separation process was monitored by Empower™ 2 Chromatography Data Software, Waters 2009 (Waters Corp., Milford, MA, USA). A mixture of MeOH p.a. (70%) and H_2_O-HPLC–Mili-Q Grade (30%) was used as a mobile phase. The total flow of the column was 1.0 mL/min, injection volume, 30 μL, column temperature, 45 °C and sample temperature, 10 °C. The detection wavelength of 210 nm was chosen. The KI methanolic solution was used for the dead time (t_D_) determination. The retention times (t_R_) were measured in minutes. The capacity factors *k* were calculated using the Empower™ 2 Chromatography Data Software according to formula *k* = (t_R_− t_D_)/t_D_, where t_R_ is the retention time of the solute, whereas t_D_ denotes the dead time obtained using an unretained analyte. Log *k*, calculated from the capacity factor *k*, is used as the lipophilicity index converted to log *P* scale. The log *k* values of the individual compounds are shown in Table 1.

### 3.3. Calculation of Lipophilicity

Log *P*, *i.e.*, the logarithm of the partition coefficient for *n-*octanol/water, was calculated using the programs CS ChemOffice Ultra ver. 10.0 (CambridgeSoft, Cambridge, MA, USA) and ACD/LogP ver. 1.0 (Advanced Chemistry Development Inc., Toronto, Canada). Clog *p* values (the logarithm of *n*-octanol/water partition coefficient based on established chemical interactions) were generated by means of CS ChemOffice Ultra ver. 10.0 (CambridgeSoft, Cambridge, MA, USA) software. The values of the individual compounds are shown in Table 1.

### 3.4. In Vitro Evaluation of AChE-Inhibiting Activity

The ability of the tested compounds to inhibit acetylcholinesterase (purchased from Sigma) from electric eel (*Electrophorus electricus* L.) was tested. The effectiveness of the inhibitor could be described by the 50% inhibitory concentration IC_50_. The IC_50_, or the half maximal inhibitory concentration, represents the concentration of an inhibitor that is required for 50% inhibition of the enzyme (sometimes it is referred to as the negative logarithm of the molar concentration inhibiting the enzyme activity by 50%, pI_50_ = log 1/IC_50_). The IC_50_ values were determined by the spectrophotometric Ellman’s method.

The IC_50_ values were determined using the spectrophotometric Ellman’s method, which is a simple, rapid and direct method to determine the SH and -S-S- group content in proteins [47]. This method is widely used for measuring of cholinesterase activity and effectivity of cholinesterase inhibitors. Cholinesterase activity is measured indirectly by quantifying the concentration of 5-thio-2-nitrobenzoic acid (TNB) ion formed in the reaction between the thiol reagent 5,5′-dithiobis-2-nitrobenzoic acid (DTNB) and thiocholine, a product of substrate (*i.e.*, acetylthiocholine, ATCh) hydrolysis by cholinesterase [48]. All tested compounds were dissolved in dioxane (concentration 0.01 M) and then diluted in demineralised water (concentration 0.001 M and 0.0001 M). The procedure of determination of IC_50_ is a slightly modified Ellman’s method according to Zdrazilova and described in detail in [49]. For determination of IC_50_ values the inhibition was determined for 25 different compound concentrations with three replicates. The obtained results are summarized in Table 1.

### 3.5. Molecular Docking

The three-dimensional structure of acetylcholinesterase, phosphonylated with sarin and complexed with the reactivator HI-6 [50], was obtained from Protein Databank (www.pdb.org, PDB code 2whq). This specific structure has been chosen due to high quality (*i.e.*, resolution of 2.15 Å), its mammalian origin (*i.e.*, mouse enzyme expressed in human cells) and the dimensions and chemical nature (*i.e.*, carbamate) of complexed inhibitor HI-6 to obtain the initial structure, reflecting the possible conformational changes, changes in the volume of the active site or its accessibility. For the molecular docking calculations, the molecules of water, molecules of crystallization and the inhibitor HI-6 were deleted, and Ser203 was dephosphonylated.

To carry out the molecular docking, GOLD 5.0 (CCDC, Cambridge, UK) in Linux version was used. The usual procedure consisting of the automatic substitution of all hydrogen atoms for the GOLD-optimized ones and the standard settings for flexible docking (free rotation and flipping of specific functional groups) were used to produce the set of optimal conformations of both the ligand and the protein. The standard GOLD scoring function was used. Prior to docking, the ligands were optimized by MM2 force field with RMS Gradient set to 0.0001 using the ChemBio3D Ultra 12.0.2.1076 (a part of ChemBioOffice Ultra 2010 Package, ver. 12.0.2.1076, CambridgeSoft, Cambridge, USA). The interaction site was defined as a cavity around Ser203 with 15 Å diameter, comprising 1566 atoms, see Figure 4A. All molecular graphics material was prepared using the Discovery Studio 3.1 Client (ver. 3.1.1.11157, Accelrys Software Inc., San Diego, CA, USA).

## 4. Conclusions

A series of twenty-five novel salicylanilide *N*-alkylcarbamates were tested for their ability to inhibit acetylcholinesterase (AChE) from electric eel (*Electrophorus electricus* L.). All the discussed compounds expressed significantly higher AChE inhibitory activity than rivastigmine and slightly lower than galanthamine. Experimental lipophilicity and other molecular descriptors of the studied compounds were determined, and the structure-activity relationships were discussed. Disubstitution by chlorine in C'_(3,4)_ of the aniline ring and the optimal length of hexyl-undecyl alkyl chains in the carbamate moiety provided the most active AChE inhibitors. Monochlorination in C'_(4)_ exhibited slightly more effective AChE inhibitors than in C'_(3)_. Generally it can be stated that compounds with higher lipophilicity showed higher inhibition, and the activity of the compounds is strongly dependent on the length of the *N*-alkyl chain. The mode of binding in the active site of AChE was investigated by molecular docking. The molecular docking yielded a series of conformations of ligands that represented quite different modes of interactions, dependent on their structure. On the basis of such orientation and the set of interactions, which were identified between the ligands and the protein residues within the gorge, it can be hypothesized that the studied compounds could act as “bulky”-blockers of the normal ionic substrate (ACh) entrance into the active site gorge.
